# Exercise makes better mind: a data mining study on effect of physical activity on academic achievement of college students

**DOI:** 10.3389/fpsyg.2023.1271431

**Published:** 2023-10-16

**Authors:** Shuang Du, Hang Hu, Kaiwen Cheng, Huan Li

**Affiliations:** ^1^College of Language Intelligence, Sichuan International Studies University, Chongqing, China; ^2^College of Teacher Education, Southwest University, Chongqing, China

**Keywords:** complex systems, college students, physical activity, running, academic performance, decision tree

## Abstract

The effect of physical activity (PA) on academic achievement has long been a hot research issue in physical education, but few studies have been conducted using machine learning methods for analyzing activity behavior. In this paper, we collected the data on both physical activity and academic performance from 2,219 undergraduate students (Mean = 19 years) over a continuous period of 12 weeks within one academic semester. Based on students’ behavioral indicators transformed from a running APP interface and the average academic course scores, two models were constructed and processed by CHAID decision tree for regression analysis and significance detection. It was found that first, to attain higher academic performance, it is imperative for students to not only exhibit exceptional activity regularity, but also sustain a reduced average step frequency; second, the students completing running exercise with an average frequency of 1 time/week and the duration of 16–25 min excelled over approximately 88 percentage of other students on academic performance; third, the processing validity and reliability of physical observation data in complex systems can be improved by utilizing decision tree as a leveraging machine learning tool and statistical method. These findings provide insights for educational practitioners and policymakers who will seek to enhance college students’ academic performance through physical education programs, combined with data mining methods.

## Introduction

The relationship between physical activity and academic performance has been studied in various adolescent populations in different countries. For instance, data from public schools in the northeastern United States confirmed a positive correlation between physical fitness test scores and pass rates in math and English course assessments (Chomitz et al., 2010). Moreover, middle school students who met the aerobic endurance running standards not only had a higher likelihood of meeting standardized test benchmarks but also demonstrated improved academic performance (Bass et al., 2013). In Spain, after controlling for BMI z-scores, waist circumference, and body fat percentage, the levels of aerobic fitness and motor skills were positively correlated with the grades on math and language tests among 6–18-year-old adolescents (Esteban-Cornejo et al., 2014). Similarly, in Japan, cardiorespiratory fitness and overall health-related fitness were found to have significant positive effect on academic performance among middle school students (Ishihara et al., 2018). Meanwhile, in a study involving 183 college students examining the relationship between physical fitness and academic performance, it was found that, apart from body mass index (BMI), all students’ physical fitness tests showed a significant positive correlation with average academic scores, indicating that high levels of physical fitness contribute positively to academic success (Başkurt et al., 2020). Zhang (2022) further investigated the factors influencing physical fitness scores among college students and identified physical fitness level, exercise frequency, and physical injuries as key factors. Currently, there is a contentious debate in the academic community regarding the apparent association between physical activity and academic performance due to varying research methodologies and data sources employed (Rodriguez et al., 2020).

In addition to the correlation and predictability of physical exercise on academic performance, some previous research has incorporated social cognitive theories from psychology to explain the underlying mechanisms. This suggests that the enhancement of students’ cognitive abilities through physical activity primarily manifests in self-control, specifically focusing on self-regulatory efficacy (Anderson et al., 2006). The impact of self-efficacy on self-regulation and its association with exercise are highlighted, with self-regulatory efficacy positively correlated with exercise intensity (Bauman et al., 2012). This explanation aligns well with social cognitive theory, as identifying oneself as an exerciser is, to some extent, influenced by past exercise experiences and serves as a source of self-efficacy (Bandura, 1997). Moreover, achieving the desired intensity of exercise is associated with various behavioral outcomes related to academic development (Strachan and Whaley, 2013), including weekly exercise minutes (Strachan et al., 2010), weekly exercise frequency, duration and intensity of vigorous exercise (Strachan and Brawley, 2008), and the number of weeks engaging in exercise (Anderson et al., 1998). These studies indicate a correlation between exercise intensity and self-regulation. Therefore, the question arises as to which specific aspect of cognitive processes in adolescents may be impacted by physical exercise and how exactly it influences cognition. Current research has only scratched the surface by exploring certain facets of cognitive processes, and the studies conducted thus far remain fragmented (Balk and Englert, 2020).

In the study of the mechanisms underlying the impact of physical activity on academic performance, two approaches are commonly used: examining the mediating variables in the causal pathway between the two factors and exploring the underlying mechanisms from other disciplines such as psychology and cognitive science. The former approach, as proposed by Kayani et al. (2018), was “physical activity → self-esteem → learning motivation and performance,” which suggests that the strongest mediator between physical activity and academic performance is self-esteem. To put it another way, physical activity could enhance students’ self-esteem, which may serve as a guarantee for their motivation and academic success. Liang and Li (2020) explored the pathway of “physical activity → physical health → academic performance” by considering both explicit physical appearance and implicit physical skills as mediating factors. The scholars underscored the pivotal role of physical fitness as a significant mediating factor influencing academic achievement (Chacón-Cuberos et al., 2020; Koçak et al., 2021). The aforementioned studies illuminate the substantial correlation existing between psychological factors, physical well-being, and academic attainment. Specifically, factors such as self-control and low self-efficacy have been found to exert a significant influence on tendencies toward overeating, weight gain, and diminished physical fitness. As the volume of data utilized in sports research continues to grow, the expansive magnitude and complex nature of sports-related data necessitate enhanced data processing techniques.

In the field of sports research, there is an increasing inclination toward the utilization of non-linear data mining techniques. These approaches offer practical insights into associations between predictor variables (e.g., team performance indicators) and dependent variables (e.g., match outcomes) (Robertson et al., 2016). Unlike linear methods, these approaches can reveal multiple patterns within the data (Mandorino et al., 2021; Teixeira et al., 2022). One widely-used non-linear method is the decision tree, which partitions samples based on maximum information entropy (Mooney et al., 2017). Hijriana and Muttaqin (2016) applied decision trees to classify academic achievement, while You et al. (2018) used them to analyze physical activity’s impact on hypertension prevention in middle-aged and older adults in China. Pei et al. (2019) evaluated five classifiers for identifying individuals with diabetes based on clinical features. Benediktus and Oetama (2020) employed the decision tree C5.0 classification algorithm, based on information entropy, to predict student academic performance and explore the role of student activeness as a predictor. The use of information entropy allows for a comprehensive exploration of intricate relationships and patterns within the complex system of physical activity (Silva et al., 2016). In this study, information entropy was also employed to construct indicators of activity patterns, with the aim of quantitatively assessing the uncertainty and randomness in the exercise patterns and trends of college students.

The progression of research involving the CHAID (Chi-squared Automatic Interaction Detector) method, in contrast to the commonly used decision tree algorithm, can be traced through multiple studies. Sanz Arazuri and de Leon Elizondo (2010) initially elucidated the application of hierarchical segmentation with CHAID, laying the foundation. Subsequently, Gómez et al. (2015) employed CHAID to pinpoint influential variables in ball screens, demonstrating its practical use. Building on this, Robertson et al. (2016) delved deeper, revealing distinctions between teams and showcasing CHAID’s effectiveness in crafting performance indicator profiles. In a more recent study, Eagle et al. (2022) extended the research by utilizing CHAID for subgroup analysis and examining its role in assessing sport-related suicide risk. Throughout these studies, CHAID consistently displayed its potential in predicting behavior indicators and elucidating causal relationships, as underscored by Schnell et al. (2014), thus emphasizing its evolving significance in the field.

In the realm of academic inquiry, a contentious debate persists regarding the connection between physical activity and academic performance. This debate stems from the diverse research methodologies and data sources employed in previous studies (Rodriguez et al., 2020). Our research endeavors to contribute to this discourse by addressing several key objectives. Firstly, we aim to unravel the intricate relationship between physical activity and academic achievement among college students. we aspire to delve deeper into the impact of physical exercise on cognitive processes in adolescents. While prior research has touched upon this topic, our goal is to identify specific facets of cognition influenced by exercise intensity. Secondly, we recognize the need for advanced data processing techniques in sports research due to the complex and expansive nature of sports-related data. By embracing non-linear data mining methodologies and leveraging information entropy, we aim to offer a fresh approach to exploring intricate relationships and patterns within the realm of physical activity and its impact on academic achievement. Furthermore, we also aim to elucidate the interplay between psychological factors, physical well-being, and academic attainment. By focusing on variables such as self-control and self-efficacy, we intend to shed light on their significant influence on behaviors related to physical fitness. Our research seeks to provide a holistic perspective on student well-being and academic success. We focused on three principal research objectives:

- Q1: Is there a correlation between the data model constructed using behavioral indicators and academic performance?
- Q2: How can effectively uncover the factors that influence academic performance and attribute interpretability to physical activity metrics through the utilization of machine learning techniques?
- Q3: How can the establishment of a pathway depicting the factors of physical activity on academic performance aid in revealing the potential mechanisms?

## Methods

### Data source and preprocessing

The research data was gathered over a continuous 12-week period during one academic semester from undergraduate students at Sichuan International Studies University in China, with an average age of 19.08 years. The data was obtained from two different systems. Firstly, approximately 9,000 academic records, including the grades of three subjects and physical fitness test scores, were retrieved from the Educational Administration System. Secondly, the physical activity log data for the research subjects during the semester was extracted from a running app installed on their mobile phones, yielding approximately 34,000 records.

In the context of this study, the log data was distributed across various business systems, necessitating a series of preprocessing steps to fully harness the data’s intrinsic value when constructing predictive indicators. Initially, the log data undergone anonymization and aggregation, involving the removal of sensitive information such as names, ID numbers, and phone numbers, followed by the correlation and integration of multiple datasets. Subsequently, common issues associated with log data, such as missing and imbalanced data, were addressed. Specifically, post-aggregation data undergone cleansing and adjustments. For instance, approximately 3.5% of students lacked running data, and there existed an imbalance in the gender ratio at college (male-to-female ratio: 1:4.3). Hence, during the preprocessing stage, missing data were addressed by eliminating invalid and duplicate records. Additionally, for datasets exhibiting skewed distributions, a Stratified Sampling approach was employed for female students to reduce the sample size, while a Bootstrap method was applied to male students to augment the sample size. This adjustment resulted in a more balanced male-to-female student data ratio of approximately 1:1.5, ensuring the integrity and validity of the predictive dataset. Ultimately, following data processing, a sample of 2,129 students was retained for the purposes of this research.

### Physical behavioral indicators

Behavioral indicators are input datasets used for machine learning modeling. Wearable sports monitoring devices or mobile apps are applied to quantify various parameters and indicators of individuals and even groups, such as movement trajectories, exercise habits, energy expenditure, and health status. There are two main types of behavioral indicators: demographic indicators and behavioral indicators. Demographic indicators include basic personal information about students, such as age, gender, and major, which have good predictive capabilities in the early stages of learning activities which represent static data (Whitener, 1989). Behavioral indicators, on the other hand, encompass changing data generated during learning activities, such as activity frequency, duration and speed. These indicators exhibit better predictive effects in the middle and later stages of activities (Hussain et al., 2018; Karthikeyan et al., 2020), representing dynamic data. The research primarily investigates students’ behavioral performance, specifically the impact of dynamic indicators on academic performance. Hence, in the construction of the analytical model, performance indicators pertaining to physical exercise are carefully chosen. Subsequently, directional indicators are employed to visually represent and classify the findings, thereby providing an effective means to elucidate the observed outcomes.

The utilization of information entropy in constructing an activity regularity indicator for college students aims to quantitatively measure the uncertainty and randomness pertaining to their exercise patterns and trends. Information entropy plays a vital role in the analysis of intricate systems in sports research, providing researchers with quantitative measures to assess and analyze various aspects of complex sports systems (Rhea et al., 2011). For instance, the utilization of entropy measurements in team sports has exhibited considerable potential in evaluating the uncertainty pertaining to players’ spatial distributions, dominant regions, and various collective team behaviors (Silva et al., 2016). Additionally, entropy has been employed to analyze the complexity and information content of heart rate variability as an indicator of activity (Namazi, 2021). In this study, entropy measures have been employed in investigating the variability of performance to unveil the underlying interactions governing activity regulation among college students, and the indicator Hx was calculated based on the distribution of exercise frequency. The entropy value was computed using the proportion of the number of exercise sessions on days for one student out of the total number of exercise sessions over days. The Hx indicator codes and descriptions are presented in Table 1.

**Table 1:** Descriptive characteristics of physical activity behavioral indicators and academic achievement indicators.

Dimension	Variable	Code	Data range	Description
Physical activity behavioral indicators	Distance covered (meters)	D_X_	592–7,413	The average distance (in meters) covered by students per running session during the 12-week semester
	Average step frequency (steps/min)	F_X_	41.7–193.1	The average frequency of running sessions per week for students during the 12-week semester
	Average running speed (meters/min)	S_X_	4.2–11.3	The average speed (in meters per minute) of students during each running session over the 12-week semester.
	Average running duration (seconds)	T_X_	328–3,415	The average duration (in seconds) of each running session for students over the 12-week semester
	Activity regularity	H_X_	0–1	The regularity of exercise $H_{X}$ was calculated based on the distribution of exercise frequency. The entropy value was computed using the proportion $f_{j}^{m}$ of the number of exercise sessions on the *jth* day for the *mth* student out of the total number of exercise sessions over *n* days. The formula for the calculation is as follows: $H_{X}=-\frac{1}{\ln\left(n\right)}\overset{n}{\underset{i=1}{\sum }}f_{j}^{m}\ln\left(f_{j}^{m}\right)$ (Formula 1)
	Activity frequency	V_X_	1–43	The total number of running sessions in the 12-week semester was determined.
Academic achievement indicators	Academic performance score (AP)	AP	23–98.7	$\mathrm{The}\;\mathrm{formula}\;for\;\mathrm{calculating}\;AP\;\mathrm{is}\;as\;\mathrm{follows}:f_{i}$ denotes the final exam score for the *ith* major-specific course, and the weight is determined based on the credit value $g_{i}$ of the course. This weight is used to calculate the weighted average score for the student’s major courses. $AP=\frac{1}{n}\overset{n}{\underset{i=1}{\sum }}\left(\frac{g_{i}}{\sum_{i=1}^{n}g_{i}}\times f_{i}\right)$ (Formula 2)

Physical behavioral indicators in current study were constructed based on the key indicators of the Physical Activity Readiness Questionnaire (PAR-Q). These indicators were developed from three aspects: exercise intensity, duration, and frequency (Thomas et al., 1992; Liang, 1994; Shephard, 2015). PAR-Q is widely used to assess physical activity levels. By scoring the three dimensions in the questionnaire, the individual’s exercise volume is calculated using the formula “intensity * duration * frequency = exercise volume.” This study built exercise indicators reflecting students’ physical activity (running) over a 12-week period in one semester. These indicators included distance covered (in meters), average step frequency (steps per minute), average pace (meters per minute), running duration (in seconds), exercise regularity, and frequency. Among them, distance, step frequency, and pace reflected exercise intensity; running duration reflected exercise time; exercise regularity and frequency reflected exercise frequency. The specific indicator codes and descriptions are presented in Table 1.

### Academic achievement indicators

Academic performance (AP) indicators, are influenced by a number of factors such as teacher subjectivity, selection bias, and student behavior (Marques et al., 2018). Scholars commonly employ standardized tests to assess AP. Examples include the Academic Aptitude Test (SAT) in the United States, the National High School Examination (ENEM) in Brazil, and the General Scholastic Ability Test (GSAT) for higher education admission in Taiwan. Some researchers also use final grades from common courses and major-specific courses within the students’ respective schools as indicators of academic performance. In the current study, the physical fitness scores and standardized average scores from major-specific courses of first-year university students over one semester were used as predictive targets to evaluate their physical fitness and academic performance. As for the selection of major-specific scores, due to the large sample size and the variation among students’ colleges and majors, AP was primarily determined by the average scores of their highest credit courses. The conversion method is detailed in Table 1.

### Data mining based on machine learning

**Figure 1:**
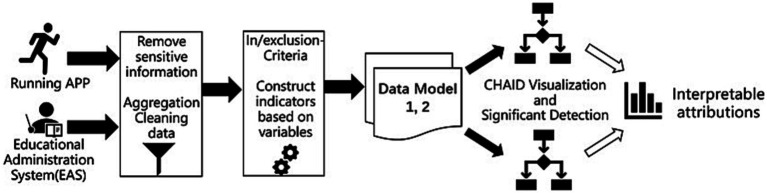
Flowchart of data mining based on physical activities.

In order to enhance the interpretability of the study’s predictions, the target variables for prediction were not the conventional classification categories such as “pass,” “good,” and “excellent,” but rather continuous variables directly associated with academic performance scores. This choice transformed the task into a typical regression problem. The study had two main parts: firstly, the data collected from the administration system and mobile apps are anonymized, aggregated, and cleaned, and the predictive variables for correlation and variance inflation factor (VIF) to identify the optimal predictors. Secondly, the CHAID decision tree algorithm was utilized for significance testing and branch prediction, providing statistical explanations and attributions to the results, and identifying potential factors influencing academic performance from the patterns of physical activity behavior among college students. The flowchart involving data collection, preprocessing, screening process, and data model construction, and CHAID decision tree modeling is shown in Figure 1.

### Data model

To validate and compare the predictive capabilities of physical behavioral indicators on academic performance, the behavioral dataset was divided into two subsets. Both subsets were associated with the predictive target variables of academic performance, forming the learner data models Model 1 and Model 2, as follows. These data models served as the data source for subsequent prediction model construction and performance comparison.

Model 1: Physical behavioral indicators (Variables) - > Academic Performance Score (All Target).

Model 2: Physical behavioral indicators (Variables) - > Academic Performance Score (Only AP > 80).

### Analysis tools

The predictive tools employed in this study utilized prediction algorithms provided by machine learning models, specifically SPSS Modeler for predictive modeling and analysis. The CHAID module in SPSS Modeler was used for decision tree visualization modeling. This module is used for branch prediction and significance analysis in the two data models. By utilizing the CHAID method, we could quickly and effectively unearth the primary influencing factors. This approach could handle nonlinear and highly correlated physical behavioral data. Furthermore, it could accommodate missing values, thus overcoming restrictions faced by traditional parametric tests in these aspects.

## Results

### Correlation analysis

Correlation analysis and variance inflation factor (VIF) tests were conducted on the behavioral indicators. The former assessed the phenomenon correlation between the predictive indicators and the target variable, while the latter evaluated the collinearity among the indicators within a controllable range. If the VIF value was less than 0.1 or greater than 10, it indicated poor predictive performance and necessitates adjustment or removal of the respective indicator (as shown in Table 2). From Table 2, it can be observed that the average running speed (S_X_) has a relatively high VIF value, but it still falls within a reasonable range. All other indicator VIF values are less than 3, indicating that all predictive indicators satisfy the collinearity condition and should be retained.

**Table 2:** Descriptive statistics, correlations, and VIF between physical behavioral indicators and academic achievement indicators.

Variable	M	SD	1	2	3	4	5	6	7	8
1. Age	19.08	0.776	–							
2. T_X_	1389.370	366.333	0.012	–						
3. D_X_	2997.380	752.255	0.041	0.875^**^	–					
4. F_X_	132.742	26.929	0.025	−0.164^**^	−0.082^**^	–				
5. S_X_	7.707	0.917	−0.063^**^	0.360^**^	−0.107^**^	−0.207^**^	–			
6. H_X_	0.649	0.136	−0.051^*^	−0.240^**^	−0.306^**^	0.040	0.085^**^	–		
7. V_X_	12.130	3.982	−0.042	−0.488^**^	−0.520^**^	0.089^**^	0.007	0.513^**^	-	
8. AP	81.944	6.210	−0.0076	−0.002	0.048^*^	−0.027	0.010	0.090^**^	0.097^**^	–
VIF	–	–	–	1.416	2.883	1.061	8.909	1.37	1.731	–

*N* = 2,129; *M*, mean; SD, stanfard deviation; VIF, variance inflation factor; *p < 0.05; **p < 0.01.

### Impact of exercise performance indicators on academic performance from data model 1

**Figure 2:**
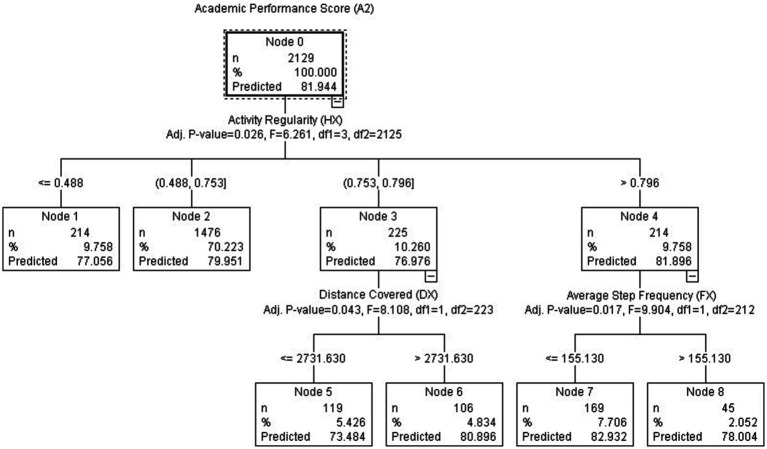
CHAID decision tree analysis diagram based on data model 1.

The analysis of academic performance was conducted based on the indicators from data model 1, as shown in Figure 2.

From Figure 2, it is evident that exercise regularity significantly influences academic performance (*p* < 0.00). In Node 2, 70% of students exhibited exercise regularity ranging from 0.488 to 0.753. These students, as long as they maintain good exercise regularity, can achieve satisfactory academic performance (AP = 79.951, comparable to the overall average of 79.553). Within the subset of students with higher exercise regularity, some individuals (Node 6) not only demonstrate regular exercise habits but also fulfill the designated running distance (Dx > 2731.63), resulting in above-average scores (AP = 80.896). The highest score is observed in Node 7, where students with the best exercise regularity (Hx > 0.796) and not necessarily fast running or high step frequency (F_X_ > 155.13) achieve the best academic performance (AP = 78.0). It is the students who exhibit regular, slower-paced, and lower step frequency exercise patterns that excel in academic performance.

### Impact of exercise performance indicators on academic performance from data model 2

**Figure 3:**
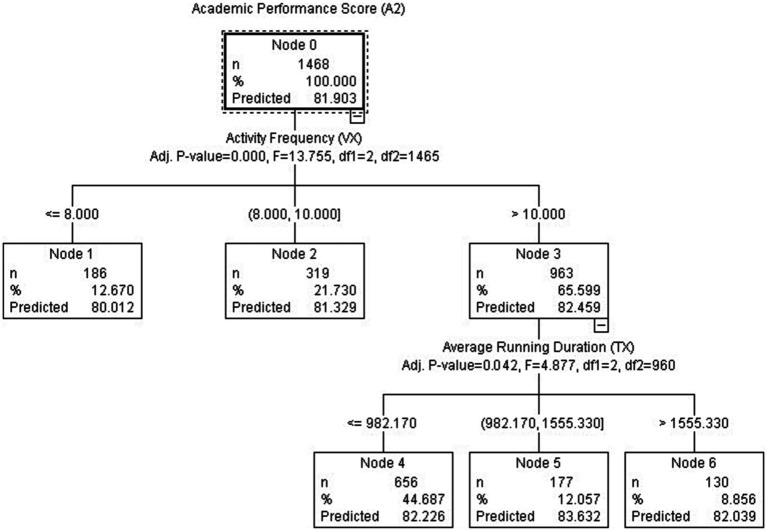
CHAID decision tree analysis diagram based on data model 2.

When investigating the impact of exercise frequency and duration on academic performance, no significant differences were found in the decision tree analysis among all study subjects (*p* > 0.05). Therefore, the study sample was reduced, focusing primarily on students with good academic performance (AP > 80). From a total of 2,129 occurrences, 1,468 individuals (accounting for 68.9%) were selected as the new sample for further analysis, as depicted in Figure 3.

Based on Figure 3, it was evident that exercise frequency had a significant impact on achieving better academic performance (*p* < 0.00). As the number of exercise sessions (V_X_) increased from 8 to 10, academic performance also increased from 80.01 to 82.46, exhibiting a linear correlation trend. Among the majority of students (65.6%), exercise frequency exceeded 10 sessions (V_X_ > 10). However, it was not the duration of each running session that determined the academic performance; instead, students (12.057%) with an average running time between 982.17 and 1555.33 s (16.4–26.1 min) achieved the best academic performance (AP = 83.632). Additionally, within this group of students, 44.69% had a running duration of less than 16 min, indicating relatively shorter running times and only meeting the minimum requirements. On the other hand, a small percentage (8.86%) of students had an average running time exceeding 26 min, indicating slower running speeds, primarily jogging or even walking, and insufficient intensity for cardiovascular exercise. Nodes 4 and Node 6 demonstrated a threshold effect, displaying an inverted U-shaped trend. While these students can also achieve satisfactory academic performance (AP > 82), their overall exercise effectiveness was inferior to that of students in Node 5, which exceeded the academic performance of approximately 88% of all other students.

## Discussion

### The effect of activity tasks on academic performance

In our academic endeavor, we undertook a correlation validation analysis to address the first research question (Q1) and utilized the CHAID methodology to identify the most substantial influencing factors in addressing the second research question (Q2). In terms of academic performance, students who successfully complete assigned tasks may achieve satisfactory average grades. However, to attain higher academic performance (AP = 82.932), as depicted in Node 7 of Figure 2, students not only need to demonstrate excellent activity regulation (H_X_ > 0.796) but also maintain a lower stride frequency (F_X_ < 155.13). This implies that students predominantly engage in jogging or walking, indicating lower exercise intensity compared to students in Node 8. It can be inferred that consistent engagement in low-intensity running promotes regular and sustained physical activity, indirectly affirming the endurance training component of exercise. This contributes to the development of students’ self-control and self-efficacy, which in turn aligns with their academic performance. In the academic domain, students are encouraged to cultivate a mindset of continuous learning and steadfastness, rather than relying solely on intense and short-term bursts of studying. It is through consistent effort and perseverance that students can build a solid foundation of knowledge and skills, enhancing their academic achievements in the long run. By integrating regular physical activity into their routines, students not only improve their cardiovascular and aerobic fitness but also develop important qualities such as discipline, focus, and resilience, all of which are conducive to academic success. This highlights the significance of maintaining a balanced approach to both physical exercise and academic pursuits, recognizing the synergistic relationship between the two domains. Therefore, emphasizing the value of consistent and moderate exercise contributes to the overall well-being and holistic development of students, ultimately benefiting their academic endeavors.

### Optimal activity frequency and duration for academic performance

During the exploration of second research question (Q2), we sought to unravel the factors that exert a substantial influence on academic performance and simultaneously imbue interpretability into the realm of physical activity metrics, leveraging the capabilities of machine learning techniques. In pursuit of this objective, we turned to the CHAID method, a powerful tool that allowed us to identify and highlight the most pivotal influencing factors. According to Figure 2, 67% of college students engage in physical activity with a frequency ranging from 7 to 14 times over the course of 12 weeks, which yields the maximum improvement in AP. Furthermore, 44.29% of students participate in physical activity between 10 and 14 times (at least once per week on average), resulting in favorable academic achievements (AP > 82). According to Figure 3, students who engage in physical activity for durations ranging from 16 to 26 min demonstrate the highest predictive capability for academic performance. Although the proportion of these students in Node 5 is not high (12.06%), it reflects the positive impact of physical activity on improving cardiorespiratory endurance and regulating self-efficacy. Considering the average running distance, most students have covered over 2 kilometers after running for 16 min, which is a critical period for cardiorespiratory/aerobic fitness (C/AF) development. These students are capable of maintaining a moderate pace during running without rushing to complete the distance task. Their awareness of self-regulation efficiency influences goal selection, persistence in goal achievement, and response to setbacks, thereby enhancing their self-regulatory abilities (Maddux et al., 2012). Allocating up to an additionally approximate half hour per day of curricular time to AP program does not affect the academic performance of primary school students negatively, even though the time allocated to other subjects usually shows a corresponding reduction.

### Mechanism underlying the impact of physical activity on academic performance

To address research question (Q3), which pertains to elucidating the potential mechanisms by which establishing pathways may be beneficial, our study furnishes evidence for a mediating pathway within the impact mechanism. Specifically, we propose the pathway as follows: “physical exercise → self-control ability → academic performance.” The self-control ability is derived from college students engaging in low-intensity running during physical exercise, which allows them to control their speed without rushing to reach their fitness goals while still achieving the required intensity. It also supports the findings of Xu et al. (2018), who concluded that executive function serves as an intermediate variable by which physical exercise promotes academic performance, explaining the pathway as “physical exercise → executive function → academic performance.” Furthermore, physical exercise offers the advantage of being regularly and consistently performed on a weekly basis, thus enhancing college students’ confidence and self-efficacy. This finding further corroborates with the research conclusion of Anderson et al. (2006) that while exercise directly influences academic performance, psychological-social factors and physical fitness levels play a mediating role. Through the expenditure of body fat calories during exercise, college students enhance their self-control ability and willpower, representing a self-regulatory structure that impacts individuals’ efforts to maintain consistency between cognition and behavior (Anderson et al., 2006).

### Data mining in sport education research

Physical activity involves complex decision-making processes, necessitating the utilization of effective tools and techniques to support physical educators. In the context of physical education research, it is essential to continuously explore the utilization of various research and experimental tools in practical investigations, fostering the in-depth application of advanced quantitative research methods and tools. In the domain of regression problems, it is imperative for machine learning algorithms to demonstrate not just robust predictive abilities, but also effective generalization. Therefore, in this study, the analysis extended beyond examining mean values of each indicator. To better capture the model’s generalization and explanatory power, the CHAID decision tree was employed, enabling statistical significance testing and offering comprehensive regression results (Morgan et al., 2013). Decision trees, as a tool in machine learning have been playing a role in researching and solving complex problems in many fields, and has gained attention as a promising approach for tackling the intricacies and uncertainties associated with analyzing physical activity. Especially in the current era of big data, the abundance of data collected from observations of physical exercise (PE) and physical activity (PA) enables the emergence of behavioral patterns. By leveraging machine learning tools and statistical methods, the processing validity and reliability of physical observation data in complex systems can be improved (Robertson and Joyce, 2015). This serves as the material foundation and underlying logic for educational data mining and data-driven approaches, which are essential for enhancing educational management and informed decision-making. For instance, unsupervised learning methods can be employed to classify or cluster groups based on sports-related data using entropy-based techniques (Rhea et al., 2011; Namazi, 2021; Yang, 2021).

## Limitations

First, this study leveraged a sizable sample for evaluating academic performance in relation to physical activity. Our research demonstrated an approach to enhance the interpretability and effectiveness of decision trees in processes. The challenges pertaining to missing physical exercise data, overfitting during model construction, and optimization of model parameters are to be addressed. Secondly, participants’ levels of physical activity may not be fully reflected in the data obtained from the running application (APP) since some special cases may have not been excluded completely, where low physical activity values could be due to student dropout or illness-related leaves and exceptionally high values could be attributed to student athletes or long-distance running enthusiasts (Lupo et al., 2017a). Thirdly, university students may engage in physical exercise for varying objectives, such as medals, participation in competitive events or improving their academic performance. Therefore, future research will delve further into the motivations behind physical exercise and their direct or indirect (mediating) impact on academic performance (Lupo et al., 2017b; Liang and Li, 2020).

## Conclusion

This study utilized machine learning methods to investigate the impact of physical activity on academic achievement among undergraduates. The decision tree model effectively captured the relationship between physical and academic performance. Activity regularity exhibited varying degrees of influence on the interaction between physical test scores and academic achievement, and explaining the relationship between physical activity and academic achievement in terms of psycho-social factors and physical fitness level. These findings contribute to the existing literature on the subject and provide insights for educational practitioners to enhance academic performance through physical activity interventions.

## Data availability statement

The original contributions presented in the study are included in the article/supplementary material, further inquiries can be directed to the corresponding author.

## Ethics statement

The studies involving humans were approved by Sichuan International Studies University. The studies were conducted in accordance with the local legislation and institutional requirements. The participants provided their written informed consent to participate in this study.

## Author contributions

SD: Formal analysis, Funding acquisition, Methodology, Writing – original draft, Writing – review & editing. HH: Supervision, Writing – review & editing. KC: Investigation, Validation, Writing – original draft, Writing – review & editing. HL: Methodology, Writing – review & editing.
